# Predictors and patterns of recurrence after curative liver resection in intrahepatic cholangiocarcinoma, for application of postoperative radiotherapy: a retrospective study

**DOI:** 10.1186/s12957-015-0637-z

**Published:** 2015-07-29

**Authors:** Eunmi Gil, Jae-Won Joh, Hee Chul Park, Jeong Il Yu, Sang Hoon Jung, Jong Man Kim

**Affiliations:** Department of Critical Care Medicine, Sungkyunkwan University School of Medicine, 50 Irowndong, Gangnam-gu, Seoul, 135-170 South Korea; Department of Surgery, Samsung Medical Center, Sungkyunkwan University School of Medicine, 50 Irwon-dong, Gangnam-gu, Seoul, 135-170 South Korea; Department of Radiation Oncology, Samsung Medical Center, Sungkyunkwan University School of Medicine, 50 Irwon-dong, Gangnam-gu, Seoul, 135-170 South Korea

**Keywords:** Intrahepatic cholangiocarcinoma, Recurrence, Hepatectomy, Adjuvant radiotherapy

## Abstract

**Background:**

The majority of patients with intrahepatic cholangiocarcinoma (IHCC) who undergo complete tumor resection subsequently develop tumor recurrence. The objectives of this study were to determine the risk factors for IHCC recurrence after curative (R0) liver resection and to identify the feasibility about postoperative adjuvant radiation therapy (RT).

**Methods:**

We retrospectively reviewed patients who underwent liver resection for IHCC between April 1995 and December 2012 at Samsung Medical Center. Cox regression analysis was performed to determine risk factors of recurrence. Patients with a recurrence in remnant liver within 2 cm from the resection margin, with or without locoregional lymph node (LN) metastases, were considered as potential RT candidates. Center-of-mass (COM) distances between the recurrent cancers and the cut surface were measured with MATLAB.

**Results:**

We included 153 out of 198 patients who underwent partial liver resection for IHCC. About two thirds (*n* = 93, 60.8 %) of patients developed recurrent disease. The median recurrence-free survival (RFS) was 14 months (range, 0–204). Tumor size ≥4.0 cm, LN metastasis and multiple tumors were significant predictors of IHCC recurrence on multivariate analysis. Tumor size ≥5.0 cm was the only factor associated with recurrence beyond the RT field in patients with recurrence. Among 93 patients with recurrence, 16 (17.2 %) patients were recurred in the RT field.

**Conclusion:**

After curative resection in IHCC, more than 60 % of patients recurred, and among recurred patients, 17.2 % were recurred within the RT field. Consequently, for control of locoregional recurrence, adjuvant RT could be carefully considered in patients with recurrence factors. Especially, patients with a tumor size larger than 5 cm should be judiciously selected for adjuvant RT.

## Background

Cholangiocarcinoma (CC) is the second most common primary hepatic malignancy, after hepatocellular carcinoma, and is divided into three general categories based on the origin of disease, intrahepatic, hilar, or distal CC [1, 2]. Although hilar CC remains the most common type, the incidence of intrahepatic cholangiocarcinoma (IHCC) is rising [3–5], and IHCC currently accounts for 20 % of all CC [5].

IHCC has a dismal prognosis with limited treatment options and a very high rate of recurrent or metastatic disease [1, 2]. Indeed, the overall mortality rate in IHCC approaches its incidence [6]. Currently, surgical resection offers the only chance for cure. However, as the disease lacks symptoms until late in its course, the majority of IHCC patients have unresectable tumors at diagnosis, and less than 50 % of patients with IHCC are surgical candidates [2, 7–11]. Even after complete resection, recurrence rates approach 52 %, with 5-year post-resection survival rates ranging from 8 to 44 %, indicating that resection alone is not sufficient for most patients [1, 6–9, 12, 13].

Repeated surgery is limited by patients’ comorbidities or by poor functional hepatic reserve. In patients with recurrence, palliative treatments such as radiation and systemic chemotherapy are the only options. However, current studies testing the role of adjuvant chemotherapy in IHCC are limited [14–16]. While adjuvant radiation therapy may improve survival in patients with microscopically positive margins, this remains controversial [17–21] and no guidelines for adjuvant therapy for IHCC exist [22]. The benefit of adjuvant RT in resected IHCC will undoubtedly depend on appropriate patient selection. The purpose of this study is to identify a patient subset at high risk for locoregional tumor recurrence after curative resection and to identify the feasibility about postoperative adjuvant radiation therapy.

## Methods

### Patient selection and data collection

We retrospectively reviewed patients 18 years of age or older with IHCC who underwent liver resection at Samsung Medical Center between April 1995 and December 2012. We excluded patients with a history of other malignancies, and those who had neoadjuvant therapy for IHCC, M1 disease, and R2 or R1 resection. Postoperatively, patients were followed with serial computed tomography (CT) scans and serial serum tumor marker (CA 19–9 and CEA) levels.

Pathology reports were reviewed for important prognostic factors including tumor size, subtype, number and histology, margin status, lymph node (LN) involvement, and the presence of lymphovascular invasion (LVI) and perineural invasion (PNI), which are known important factors for tumor recurrence and patient survival [23–25]. Satellite nodules were considered as multiple tumors. Patients who did not undergo LN dissection were regarded as node-negative, as this is consistent with clinical practice patterns. The first postoperative follow-up evaluation was usually 1 month after the operation, and subsequent visits were performed according to the surgeon’s discretion. Overall survival was ascertained through the clinical follow-up documented in each patient’s medical record.

### Recurrence classification

The regions of the recurrent cancers and the cut surface were delineated on the follow-up CT or magnetic resonance (MR) using Eclipse ver. 10.0 (Varian, Palo Alto, CA), and the structure files in digital imaging and communications in medicine (DICOM), including the regions, were exported to an in-house program in MATLAB (Mathworks, Natick, NA). The closest, farthest, and center-of-mass (COM) distances between the recurrent cancers and the cut surface were measured with the program. Curability was determined according to the R-classification of the International Union Against Cancer as R0, no residual tumor; R1, presence of microscopic disease despite removal of all identifiable tumor; and R2, tumor left macroscopically in situ.

Regional LNs of IHCC included the hilar LNs. And according to LN drainage, tumors in the right liver (segments 5–8) included pancreaticoduodenal LNs, and tumors in the left liver (segments 2–4) included gastrohepatic LNs. Diseases that spread to the celiac, periaortic, or caval LNs were usually considered distant metastases (M1 node), but clinical radiation therapy (RT) fields generally encompassed periaortic or caval LNs around celiac LNs.

**Fig. 1:**
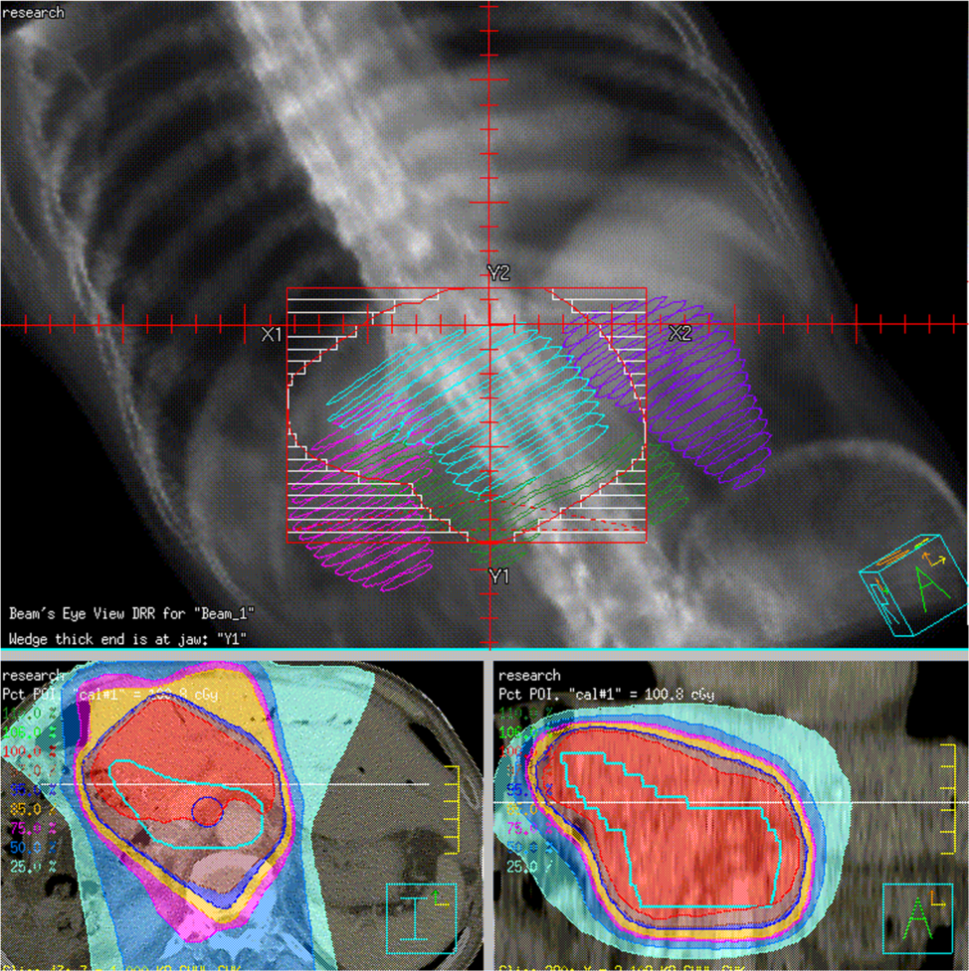
Hypothetical adjuvant radiotherapy fields in intrahepatic cholangiocarcinoma. Potential clinical target volume (CTV) is defined as remnant liver within 1 cm from resection margin, perihilar, periduodenal, peripancreatic, celiac, caval, and periaortic (from 1 cm above of celiac axis to left renal artery bifurcation) lymph node area

Therefore, local recurrence in the present study was defined as any recurrence within the potential planning target volume (PTV) in RT fields. We defined the potential clinical target volume (CTV) in the present study as remnant liver within 1 cm from resection margin, perihilar, periduodenal, peripancreatic, celiac, caval, and periaortic (from 1 cm above of celiac axis to left renal artery bifurcation) LN area. PTV was decided with an additional 1 cm margin for setup and respiration uncertainties. We assumed radiation delivered as a 3-D conformal technique, and the remnant liver, kidneys, stomach, duodenum, other bowel, and spinal cord were considered as organs at risk. Hypothetical CTV, RT fields were displayed in Fig. 1. And, the authors hypothesized patients who recurred in this area which is remnant liver within 2 cm of the COM with or without locoregional LN metastasis as potential RT candidates.

### Statistical analysis

Summary statistics are reported as total and percentage for categorical variables and as median values and range for continuous variables. Survival and recurrence rates were calculated by the Kaplan–Meier method. Cox regression analysis was performed to determine recurrence factors. If data for a variable were missing in >10 % of cases, the variable was not used in the analysis. Factors significant at a level of *p* < 0.1 were included in the multivariate analysis. A receiver operating characteristics (ROC) curve was used to identify optimal cutoff points for each marker. All statistical analyses were carried out using IBM SPSS statistics version 22. A *p* value of less than 0.05 was considered to be statistically significant.

## Results

### Patients and tumor characteristics

**Fig. 2:**
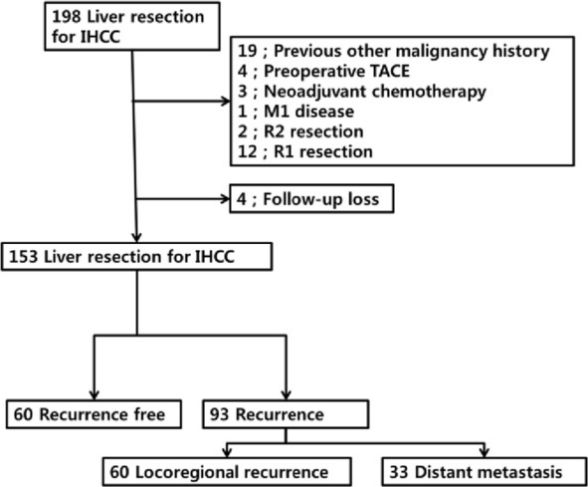
Patient selection and recurrence after liver resection for intrahepatic cholangiocarcinoma

A total of 198 patients underwent partial liver resection for IHCC between April 1995 and December 2012. We excluded 19 patients who had another previous malignancy, four patients who had preoperative transarterial chemoembolization (TACE), three patients who had neoadjuvant chemotherapy before hepatectomy, a patient with bone metastasis who underwent palliative hepatectomy, two patients with R2 disease, and 12 patients with R1 disease. Four patients were lost to follow-up (Fig. 2).

The final study population (*n* = 153) had resected IHCC only, with a median age of 59 years (range, 37–80 years); 96 patients (62.7 %) were male. Operative and pathologic variables are summarized in Table 1. The majority (90.1 %) of patients required a hemihepatectomy, extended resection, or multiple segmental resection. Eighty-one patients (52.9 %) underwent lymphadenectomy. The median tumor size of the dominant lesion was 4.5 cm (range, 0.5–14.0 cm). LN metastasis occurred in 38 patients (24.8 %), and 37 patients (24.2 %) had multiple confirmed tumors in the final pathologic report. One hundred seventeen patients (76.5 %) had the mass-forming subtype of IHCC, while 36 patients (23.5 %) had the periductal infiltrating type or combined both types. LVI was found in 35.9 % of patients, and perineural invasion (PNI) was found in 23.5 %. Two patients had adjuvant chemoradiation therapy after curative resection.

**Table 1:** Clinicopathologic characteristics of patients with intrahepatic cholangiocarcinoma (*n* = 153)

Variable	Patients, *n* (%)	Median (range)
Age		59 (37–80)
Male	96 (62.7)	
Operative characteristics		
Type of resection		
Wedge resection	3 (2.0)	
Segmentectomy	11 (7.2)	
Multiple segmentectomy	25 (16.3)	
Hemihepatectomy	92 (60.1)	
Extended hemihepatectomy	22 (14.4)	
Pathologic characteristics		
Tumor size, cm		4.5 (0.5–14.0)
Tumor size ≥4 cm	97 (63.4)	
Multiple tumors	37 (24.2)	
Lymphadenectomy performed	81 (52.9)	
LN positive disease	38 (24.8)	
Number of LN retrieved		7 (1–44)
Number of positive nodes		0 (0–22)
Subtype		
Mass forming	117 (76.5)	
Periductal infiltrative	36 (23.5)	
Histology		
Adenocarcinoma	140 (91.5 %)	
Non-adenocarcinoma	13 (8.5 %)	
Lymphovascular invasion	55 (35.9)	
Perineural invasion	36 (23.5)	

*LN* lymph node

### Recurrence and survival

**Fig. 3:**
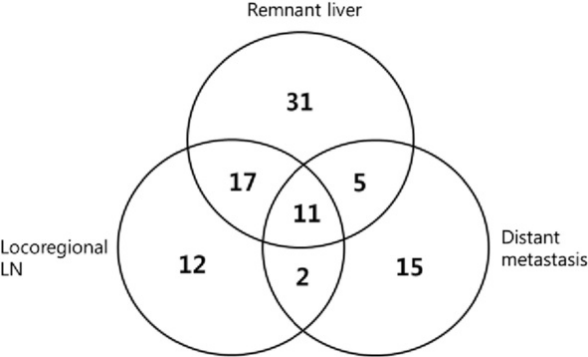
Recurrence pattern of intrahepatic cholangiocarcinoma (*n* = 93)

After a median follow-up of 21 months (range, 1–204 months), recurrence had developed in 93 patients (60.8 %) (Fig. 2). Among 93 patients with recurrence, 60 patients (64.5 %) had recurrences in the remnant liver and/or locoregional LNs. Thirty-three patients (35.5 %) were diagnosed with distant metastasis. The most common distant metastasis site was the lung. Eleven patients recurred in the liver, locoregional LN, and distant metastasis sites. Figure 3 shows recurrence patterns of IHCC. The median recurrence-free survival (RFS) was 14 months (range, 0–204), and the median overall survival (OS) was 35 months (range, 1–204). Tumor size was an important factor for recurrence based on univariate Cox regression analysis (*p* < 0.001). LN metastasis, tumor subtype, multiple tumors, LVI, PNI, and prothrombin time–international normalized ratio (PT–INR) were recurrence factors based on univariate analysis. The optimal cutoff for tumor size as a recurrence risk factor was ≥4.0 cm (*p* < 0.001 AUC 0.740, CI 0.661–0.819). Tumor size ≥4.0 cm (*p* < 0.001), LN metastasis (*p* = 0.006), and multiple tumors (*p* = 0.015) were significant predictors of IHCC recurrence on multivariate analysis (Table 2). Predictors of survival were similar to those for recurrence. On univariate analysis, INR, tumor subtype, tumor size, multiple tumors, LVI, PNI, and LN metastasis were risk factors. Tumor size ≥4.0 cm (*p* < 0.001), LN metastasis (*p* < 0.01), multiple tumors (*p* = 0.014), and LVI (*p* = 0.015) were risk factors on multivariate analysis (Table 3).

**Table 2:** Predictors of recurrence after resection of intrahepatic cholangiocarcinoma (*n* = 153)

Characteristic	Univariate		Multivariate	
	HR (95 % CI)	*p* value	HR (95 % CI)	*p* value
Sex	1.046 (0.688–1.589)	0.835		
Age	1.011 (0.988–1.034)	0.360		
PT–INR	7.894 (1.041–59.841)	0.046		
Type of resection		0.483		
Wedge resection	0.917 (0.223–9.767)	0.904		
Segmentectomy	1.264 (0.601–2.661)	0.537		
Multiple segmentectomy	0.729 (0.399–1.335)	0.306		
Hemihepatectomy	1			
Extended hemihepatectomy	1.419 (0.799–2.518)	0.232		
Tumor subtype	0.386 (0.221–0.676)	0.001		
Histology	0.394 (0.144–1.075)	0.069		
Tumor size ≥4.0 cm	2.932 (1.830–4.696)	<0.001	2.522 (1.556–4.088)	<0.001
Multiple tumors	2.020 (1.279–3.191)	0.003	1.815 (1.125–2.927)	0.015
LVI	1.866 (1.232–2.826)	0.003		
PNI	1.825 (1.156–2.880)	0.010		
LN metastasis	2.277 (1.424–3.642)	0.001	2.004 (1.221–3.289)	0.006
Resection margin	0.988 (0.972–1.044)	0.144		
HBV	1.135 (0.685–1.881)	0.623		
HCV	1.550 (0.624–3.849)	0.345		

*LVI* lymphovascular invasion, *PNI* perineural invasion, *HBV* hepatitis B virus, *HCV* hepatitis C virus, *PT–INR* prothrombin time–international normalized ratio

**Table 3:** Predictors of survival after resection of intrahepatic cholangiocarcinoma (*n* = 153)

Characteristic	Univariate		Multivariate	
	HR (95 % CI)	*p* value	HR (95 % CI)	*p* value
Sex	0.873 (0.568–1.344)	0.538		
Age	1.011 (0.988–1.036)	0.347		
PT–INR	24.618 (3.449–161.735)	0.001		
Type of resection		0.137		
Wedge resection	0.450 (0.062–3.258)	0.429		
Segmentectomy	0.949 (0.432–2.087)	0.897		
Multiple segmentectomy	0.655 (0.343–1.252)	0.201		
Hemihepatectomy	1			
Extended hemihepatectomy	1.677 (0.969–2.903)	0.065		
Tumor subtype	0.367 (0.207–0.654)	0.001	0.536 (0.297–0.968)	0.039
Histology	0.680 (0.314–1.475)	0.329		
Tumor size ≥4.0 cm	3.162 (1.928–5.184)	<0.001	2.539 (1.512–4.264)	<0.001
Multiple tumor	2.475 (1.585–3.864)	<0.001	1.782 (1.122–2.828)	0.014
LVI	2.299 (1.520–3.475)	<0.001	1.684 (1.107–2.564)	0.015
PNI	1.869 (1.181–2.957)	0.008		
LN metastasis	3.902 (2.499–6.091)	<0.001	3.567 (2.240–5.680)	<0.001
Resection margin	0.991 (0.974–1.008)	0.294		
HBV	1.165 (0.695–1.955)	0.562		
HCV	1.226 (0.496–3.026)	0.659		

*LVI* lymphovascular invasion, *PNI* perineural invasion, *HBV* hepatitis B virus, *HCV* hepatitis C virus, *PT–INR* prothrombin time–international normalized ratio

### Potential adjuvant RT candidates

**Fig. 4:**
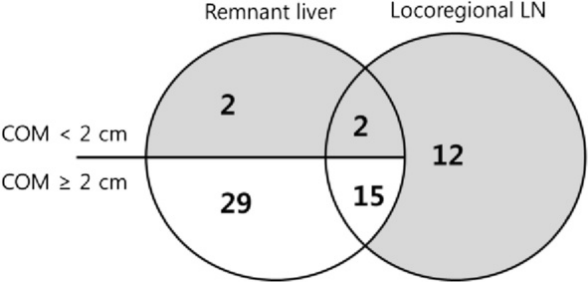
Recurrence in remnant liver and/or locoregional LNs of intrahepatic cholangiocarcinoma (*n* = 60). Patients in *gray area* (*n* = 16) included hypothetical radiation therapy field

Figure 3 shows recurrence in remnant liver and/or locoregional LNs of IHCC. Among 93 patients with recurrence, 60 (64.5 %) recurred in remnant liver and/or locoregional LNs and only 16 patients (17.2 %; gray area in Fig. 4) were included in the hypothetical RT field. Forty-four patients recurred in remnant liver more than 2 cm far from the cut surface with or without locoregional LN metastasis, in other words, recurred beyond the RT field (white area in Fig. 4). Among the 16 patients, two patients recurred in the liver, 12 recurred in locoregional LNs, and two patients recurred in both the liver and locoregional LNs. Among patient recurred, tumor size (*p* < 0.001) was the only predictive factor of recurrence beyond the hypothetical RT field. The optimal cutoff point for tumor size as a predictor of recurrence beyond the hypothetical RT field was ≥5.0 cm (*p* = 0.045 AUC 0.628, CI 0.504–0.751) (*n* = 93) (Table 4). There were no statistically significant factors between four patients recurred in remnant liver within 2 cm of COM and other recurred patients.

**Table 4:** Predictors of recurrence beyond the radiation therapy field in recurrent patients (*n* = 93)

Characteristic	HR (95 % CI)	*p* value
Sex	1.194 (0.745–1.914)	0.462
Age	0.976 (0.948–1.004)	0.095
PT–INR	7.155 (0.498–102.760)	0.148
Tumor subtype	0.566 (0.294–1.092)	0.090
Histology	0.753 (0.234–2.426)	0.635
Tumor size	1.204 (1.101–1.317)	<0.001
Tumor size ≥5.0 cm	3.140 (1.890–5.217)	<0.001
LVI	1.288 (0.810–2.049)	0.284
PNI	1.239 (0.745–2.060)	0.409
LN metastasis	1.567 (0.924–2.655)	0.095
Resection margin	0.985 (0.968–1.004)	0.113
HBV	0.806 (0.448–1.450)	0.471
HCV	1.236 (0.496–3.085)	0.649

*LVI* lymphovascular invasion, *PNI* perineural invasion, *HBV* hepatitis B virus, *HCV* hepatitis C virus, *PT–INR* prothrombin time–international normalized ratio

## Discussion

Surgical resection is the only potential cure for IHCC. Although resectability of IHCC has improved recently [10], the success rate remains poor [10, 11]. Even in patients who are able to undergo surgical resection, positive margins are common [10, 11]. The use of R0 resection provides the best chance for cure but is only achievable in about 30 % of patients at presentation [11]. Studies have demonstrated that patients who had an R1 resection had a survival similar to those who were treated palliatively (5 vs. 7 months) and that local recurrence is a major problem in patients with R1 resections [11, 26].

Even after R0 resection, many studies reported recurrence rates more than 50 %, representing that resection alone is not sufficient for most patients [1, 6–9, 12, 13]. Like other studies, our data shows 60.8 % recurrence rate in IHCC after R0 resection. In patients with recurrence, palliative treatments such as radiation and systemic chemotherapy could be considered. Earlier reports of postoperative adjuvant therapy for the treatment of biliary tract carcinoma have indicated the potential usefulness of radiotherapy and chemotherapy [27–30]. However, current studies testing the role of adjuvant chemotherapy in IHCC are limited [14–16]. While adjuvant radiation therapy may improve survival in patients with microscopically positive margins, this remains controversial [17–21]. Moreover, nearly all these studies were based on cases of noncurative resection and were experimental reports rather than clinical trial reports. In addition, some hilar CC have been misclassified as IHCC, thereby affecting analysis [21]. Thus, the benefit of adjuvant therapy for carcinoma of the biliary tract has not yet been clarified, and no guidelines for adjuvant therapy for IHCC exist [22]. The benefit of postoperative adjuvant RT will depend on appropriate patient selection. So, authors determined to identify a patient subset at high risk for locoregional tumor recurrence after R0 resection and a subgroup among these patients who could be covered within the RT field.

As mentioned above, some hilar CC have been misclassified as IHCC and many studies included R1 resection also. However, this report included only patients with pathologically confirmed IHCC with R0 resection. Patients with perihilar or distal CC, history of other malignancy, history of neoadjuvant therapy (TACE and chemotherapy), M1 disease, and R1 and R2 diseases were excluded from this analysis. After curative resection, two patients had adjuvant chemoradiation therapy. A patient was 53 years old, female, and with multiple IHCC. The maximum tumor size was 5.5 cm. IHCC recurred after 15 months of surgery. Another patient was 48 years old, female, and with multiple IHCC. The maximum tumor size was 7.8 cm, and LN metastasis exists during surgery. IHCC recurred after a month, and she died after 4 months of surgery. Because only two patients had adjuvant treatment, it was impossible to analyze statistical significance. And after IHCC recurrence, 12 patients had palliative chemoradiation therapy and a patient had palliative RT. There was no significant difference about survival between two groups that had palliative treatment or not (data not shown).

Jarnagin et al. [31] reported about adjuvant treatment after surgery of extrahepatic cholangiocarcinoma. After surgery, locoregional recurrence is a predominant pattern of failure even with R0 resection, and as local failure can be associated with liver failure and death, this argues for adjuvant radiation and chemoradiation therapy to prevent local recurrence. Shinohara et al. [21] reported radiation used adjuvantly with surgery was associated with an improved median survival compared with radiation alone and was associated with a 9.3 % reduction in the risk of death. However, although radiation did prolong survival in these patients, it did not appear to cure their disease. And these studies did not specify the criteria about adjuvant treatment candidates after curative resection. The benefit of adjuvant RT in resected IHCC will depend on appropriate patient selection.

Pathologic factors that predict increased rates of recurrence and worse outcomes include multiple hepatic tumors, nodal involvement, and vascular invasion, which was reflected in the current American Joint Committee on Cancer (AJCC) 7th edition staging system [8, 32]. Although LN stats have been strongly associated with prognosis in almost all studies [33–35], data on tumor size have been more disparate, with some studies finding no effect of tumor size [36–38] and other larger studies showing that size affected survival [33–35]. Previously, the failure to identify an effect of tumor size on OS may be explained by the limited number of patients with small (e.g., <5 cm) tumors included in many prior studies. Our study found a tumor size larger than 4 cm is an important risk factor of recurrence and survival. Moreover, tumor size larger than 5 cm is the only risk factor of recurrence beyond the hypothetical RT field. Previous studies did not consider about tumor size as an indication of adjuvant treatment. Future studies about adjuvant RT and the AJCC staging system may need to reconsider the impact of tumor size on prognosis and should include tumor size as selection criteria of adjuvant treatment.

Our findings should be interpreted in view of certain limitations. First, retrospective study and the sample size were small. Moreover, this study was accrued over a long period (1995–2012), while both diagnostic and therapeutic procedures for IHCC have changed notably over these years. There may be a selection bias in how patients were chosen for surgical therapy. Therefore, further multicentric prospective and randomized clinical trials are needed to determine the usefulness of adjuvant RT to control locoregional recurrence after R0 resection.

With IHCC, NCCN recommendations include observation for R0 resection or some type of adjuvant therapy for R1 or R2 resections. However, this study demonstrates that tumor recurrence is common (60.8 %) after complete resection (R0) of IHCC. In this light, after R0 curative liver resection, observation is not appropriate. For control of recurrence, aggressive adjuvant treatment should be considered even if curative resection is achieved. In our study, tumor size ≥4.0 cm, LN metastasis, and multiple tumors predicted recurrence after curative resection. Among recurred patients, locoregional recurrence covered in the hypothetical RT field occurred less than 20 %. Even though a small portion of patients recurred in hypothetical RT field, authors consider postoperative RT might be a useful adjuvant modality because RT could sterilize the field; as a result, it could lead to lower the recurrence rate by lowering in and out field recurrence rate.

The benefit of adjuvant therapy in resected IHCC will depend on appropriate patient selection. Consequently, for producing positive effect about adjuvant RT after R0 resection in IHCC, RT candidates should be carefully selected in patients with recurrence factors, especially patients with a tumor size larger than 5 cm.

## Conclusion

For cholangiocarcinomas, the tumor location and resectability remain prime factors in disease control, whereas adjuvant chemotherapy and radiation remain more controversial. However, surgical resection alone is not sufficient for IHCC despite curative resection. We suggest that patients with tumor size larger than 4 cm, LN metastasis, and multiple tumors could be considered about receiving adjuvant RT for control of locoregional recurrence after R0 resection. However, patients with a tumor size larger than 5 cm should be carefully selected as adjuvant RT candidates. Further studies investigating the subset of high-risk recurrence groups as well as optimal adjuvant therapy for IHCC are needed.
